# The contribution of the ^1^H-MRS lipid signal to cervical cancer prognosis: a preliminary study

**DOI:** 10.1186/s41747-022-00300-1

**Published:** 2022-10-03

**Authors:** Miriam Dolciami, Rossella Canese, Claudia Testa, Angelina Pernazza, Giusi Santangelo, Innocenza Palaia, Carlo Della Rocca, Carlo Catalano, Lucia Manganaro

**Affiliations:** 1 Department of Radiological, Oncological and Pathological Sciences, Policlinico Umberto I, Sapienza University of Rome, Rome, Italy; 2 Core Facilities, Istituto Superiore di Sanità, Viale Regina Elena 299, 00161 Rome, Italy; 3 Department of Physics and Astronomy, University of Bologna, Bologna, Italy; 4 Department of Maternal and Child Health and Urological Sciences, Umberto I Hospital, “Sapienza” University of Rome, Rome, Italy

**Keywords:** Choline, Lipids, Magnetic resonance imaging, Proton magnetic resonance spectroscopy, Uterine cervical neoplasms

## Abstract

**Background:**

The aim of this study was to investigate the role of the lipid peak derived from ^1^H magnetic resonance (MR) spectroscopy in assessing cervical cancer prognosis, particularly in assessing response to neoadjuvant chemotherapy (NACT) of locally advanced cervical cancer (LACC).

**Methods:**

We enrolled 17 patients with histologically proven cervical cancer who underwent 3-T MR imaging at baseline. In addition to conventional imaging sequences for pelvic assessment, the protocol included a single-voxel point-resolved spectroscopy (PRESS) sequence, with repetition time of 1,500 ms and echo times of 28 and 144 ms. Spectra were analysed using the LCModel fitting routine, thus extracting multiple metabolites, including lipids (Lip) and total choline (tCho). Patients with LACC were treated with NACT and reassessed by MRI at term. Based on tumour volume reduction, patients were classified as good responder (GR; tumour volume reduction > 50%) and poor responder or nonresponder (PR-or-NR; tumour volume reduction ≤ 50%).

**Results:**

Of 17 patients, 11 were LACC. Of these 11, only 6 had both completed NACT and had good-quality ^1^H-MR spectra; 3 GR and 3 PR-or-NR. A significant difference in lipid values was observed in the two groups of patients, particularly with higher Lip values and higher Lip/tCho ratio in PR-NR patients (*p* =0.040). A significant difference was also observed in choline distribution (tCho), with higher values in GR patients (*p* = 0.040).

**Conclusions:**

Assessment of lipid peak at ^1^H-MR spectroscopy could be an additional quantitative parameter in predicting the response to NACT in patients with LACC.

**Supplementary Information:**

The online version contains supplementary material available at 10.1186/s41747-022-00300-1.

## Key points

- ^1^H magnetic resonance spectroscopy could predict therapy response in locally advanced cervical cancer (LACC).
- High lipid signal and lipids/choline ratio correlated with poor prognosis in patients with LACC.
- Lipid signal can be considered a new noninvasive prognostic biomarker in patients with LACC.

## Background

Uterin cervical cancer (CC) is the fourth most common malignancy and cancer-related mortality cause in women worldwide, with a higher prevalence in developing countries [1]. Although CC incidence has been reduced by human papilloma virus screening and vaccination programs, GLOBOCAN still estimates more than half a million new cases per year and more than 300,000 deaths per year [2, 3].

Revised and updated in 2018, the International Federation of Gynecology and Obstetrics (FIGO) classification is the most widely used for staging cervical cancer [4]. As for treatment, surgery represents the gold standard in early-stage CC. However, a significant portion of these patients are already diagnosed as locally advanced cervical cancer (LACC), *i.e.*, FIGO 2018 stage from IIb to IVa, thus requiring combined therapies, such as concurrent chemo-radiotherapy or neoadjuvant chemotherapy combined with radical surgery [5].

Due to its excellent soft-tissue characterisation, magnetic resonance imaging (MRI) represents the imaging technique of choice for the staging of CC, playing a fundamental role in assessing therapeutic strategy and response to therapy [6, 7]. Besides the anatomical characterisation provided by conventional MRI, magnetic resonance offers functional multiparametric approaches in clinical oncology to improve *in vivo* tissue characterisation [8, 9]. Among them, localised proton magnetic resonance spectroscopy (^1^H-MRS) allows *in vivo* metabolic characterisation of a body district or a suspicious lesion giving noninvasive information useful for a more accurate and faster diagnosis [10–12].

For more than two decades, *in vivo* MRS has been widely used to investigate lesions at different anatomical sites [13–15]. Technical difficulties due to artifacts caused by respiratory movements, bowel peristalsis and possible contamination of the signal by pericervical fat have limited the use of MRS in the evaluation of the abdomen and pelvis in the past.

In recent years, technical improvements of the MRI systems and software have allowed the extension of ^1^H-MRS investigation to the pelvis and its pathologies, including CC. In a first study, women with known CC revealed strong resonances (large peaks) corresponding to total choline (tCho), whereas normal controls showed spectral peaks corresponding to lipids (Lip) and creatine (Cr) [16]. A study by Booth et al. investigated the role of *in vivo*^1^H-MRS as a useful tool to distinguish between benign and malignant gynaecologic lesions, including CC. They focused on the evaluation of the tCho signal, but no difference was observed in this peak between benign and malignant forms [17]. A recent study performed at 7 T found ^1^H-MRS useful in differentiating the CC grading by estimating the level of unsaturation of fatty acid analysing the lipid signal 2.1/1.3 ppm ratio. In particular, poorly differentiated tumours showed more fatty acid unsaturation [18]. Although interesting, this study is limited to the high-field magnet, which is not common in clinical settings.

Using the most popular MRI systems (1.5 and 3 T) with less signal and spectral resolution, it is difficult to quantify the signal at 2.1 ppm and distinguish it from total *N*-acetyl-aspartate (NAA), which has been observed in breast, cervical, prostate and ovarian cancer and is assigned to the −CH_3_ moiety of sialic acid or *N*-acetyl groups of glycoproteins [19]. Studies combining *in vivo* end *ex vivo*^1^H-MRS found that the presence of the −CH_2_ signal *in vivo* (resonating at 1.28 ppm) predicted the presence of cancer with a sensitivity of 77% and a specificity of 94% [20]. The role of ^1^H-MRS as a response biomarker in CC has also been investigated, but with uncertain results [21, 22]. Up to now, ^1^H-MRS has shown limited results in the assessment of CC, and to the best of our knowledge, no study has investigated the role of the lipid peak in predicting response to therapy in LACC.

The aim of this study was to investigate the prognostic contribution of a quantitative ^1^H-MRS protocol, specifically the role of the lipid peak, in assessing response to neoadjuvant therapy in LACC.

## Methods

### Patients

The study was approved by the Departmental Ethical Committee, and written informed consent was obtained from all individuals before participation. Patients aged from 18 to 5 years with histopathologically confirmed primary cervical carcinoma and clinical or ultrasound suspicion of locally advanced disease were included in the study. Any patient who had undergone prior surgery or chemotherapy or had a concurrent other tumour was excluded. Between December 2019 and July 2021, 17 patients with histopathologically proven cervical cancer and suspected locally advanced disease underwent pelvic MRI examination, with the addition of a ^1^H-MRS sequence as part of the protocol. Two radiologists with respectively 25 (L.M.) and 5 (M.D.) years of experience in female pelvis imaging, evaluated in consensus the baseline MRI examination and staged the patients according to FIGO 2018 classification [4]. Of 17 patients, 11 were evaluated to have a LACC and treated with neoadjuvant chemotherapy (3 cycles of cisplatin 75 mg/mq and taxol 175 mg/mq). At treatment completion, patients were revaluated (follow-up MRI) and the two same radiologists (L.M., M.D.) assessed in consensus the percentage of tumour volume reduction. Patients were then divided into two groups: good responders (GR) if tumour volume reduction was > 50% and poor responders or nonresponders (PR-or-NR) if reduction was ≤ 50%.

### MRI and ^1^H-MRS protocol

Images were acquired using a superconducting magnet operating at 3 T (Discovery MR 750; General Electric Healthcare, Milwaukee, WI, USA) with a 32-channel phased-array coil positioned on the lower abdomen. In order to have moderate bladder filling, patients were asked to urinate one hour before the examination. All subjects entered the magnet in the supine position, using the feet-first mode. Prior to the examination, 20 mg of hyoscine *N*-butyl bromide (Buscopan; Boehringer-Ingelheim, Ingelheim, Germany) was injected intravenously to reduce bowel movements.

The imaging included the following sequences: sagittal, paracoronal and paraaxial T2-weighted fast spin-echo, the latter two oriented parallel to the long axis and short axis of the cervical canal, respectively; paraaxial T1-weighted fast spin-echo, with and without fat saturation; paraaxial diffusion-weighted imaging with *b* values of 0, 500, and 1,000 s/mm^2^ to obtain apparent diffusion coefficient maps; axial T2-weighted fast spin-echo from the renal hila to the pubic symphysis. Scanning parameters are shown in Table 1.

**Table 1:** Magnetic resonance imaging protocol

Sequence	Scan planes	TR/TE (ms)	FOV (mm)	NEX	Matrix	Slice thickness (mm)	Interslice gap (mm)
FSE	Sagittal	3,411/121	320 x 320	2	320 x 224	4	1
T2-weighted FSE	Paraaxial, paracoronal	5,089/127	240 x 240	2	320 x 224	4	1
T1-weighted FSE	Paraaxial	400/10	240 x 240	2		4	1
DWI	Paraaxial	2,000/57	240 x 240	2	160 x 80	3.5	0
T2-weighted FSE	Axial	5,000/125	310 x 310	2	320 x 224	4	1

*b* values DWI were 0, 500, and 1,000 s/mm^2^

*DWI* Diffusion-weighted imaging, *FA* Flip angle, *FOV* Field of view, *FSE* Fast spin-echo, *NEX* Number of excitations, *TE* Echo time, *TR* Repetition time

^1^H-MRS was acquired using a single-voxel point resolved spectroscopy (PRESS) sequence, with repetition time (TR) of 1,500 ms and echo times (TE) of 28 and 144 ms. As indicated by the most experienced radiologist (L.M.), for each subject, the voxel (average volume 18 × 18 × 18 mm^3^) was positioned in the central part of the tumour, carefully avoiding contamination from surrounding tissues and areas of necrosis, using as reference the T2-weighted images acquired along the three orthogonal planes. The shorter TE (28 ms) was chosen for metabolite quantification, while the longest TE (144 ms) was chosen for the detection of lactate (Lac) whose signal can be hidden when a large lipid signal resonating at 1.28 ppm is also present. Automated shimming using the scanner software was sufficient to acquire good quality spectra (linewidth 9 ± 5 Hz, mean ± standard deviation), thus reducing the total acquisition time.

The water signal was suppressed by using a presequence composed by chemical selective saturation, CHESS, pulses followed by crusher gradients. Thirty-two excitations were sufficient to acquire metabolite spectra from voxels ranging between 2.7 and 6.2 mL with sufficient signal-to-noise-ratio. Unsuppressed water signal was acquired from the same voxel with the same parameters except for a reduced number of excitations (1 instead of 32).

The total scan time, including MRI and ^1^H-MRS, was about 28 min, with the latter lasting less than 5 min.

### Data analysis

Spectra were analysed using LCModel fitting routine that calculates the best fit to the experimental spectrum as a linear combination of model spectra (*i.e.*, metabolite solutions) [23]. Seventeen metabolites were included in the basis set: alanine (Ala), aspartate (Asp), creatine (Cr), glucose (Glc), glutamate (Glu), glutamine (Gln), glutathione (GSH), glycine (Glyc), glycerophosphorylcholine (GPC), phosphorylcholine (PCho), myoinositol (Ins), Lac, *N*-acetylaspartate (NAA), *N*-acetylaspartylglutamate (NAAG), phosphocreatine (PCr), scyllo-inositol and taurine (Tau). Spectra of lipids and macromolecules were also included in the basis set. The J-coupling modulation experienced by Ins, Glu, and Gln signals was automatically accounted for in the LCModel basis sets. Only metabolites that were estimated to have Cramer-Rao lower bounds lower than 20%, which corresponded to an estimated concentration error lower than 0.2 μmol/g, were included in the quantitative analysis. In some cases, metabolites with overlapping or very close resonance are given as their sum (for example tCho = GPC + PCho and Glx = Glu + Gln).

The present protocol refers the metabolite signal intensity to the water signal. Corrections for the number of protons present in each resonance, assuming an 80% water concentration, are also included and allows for a reasonable estimation in mmol/L (mM units) [24]. The complete quantitative protocol should also include the measurements of T2 and T1 relaxation times, but the necessity to reduce acquisition time as much as possible during patient analyses made it preferable to have a rough estimate for metabolite quantification.

### Histopathology

Before MRI/1H-MRS examination, all patients underwent cervical biopsy for diagnostic purposes, and we reported histopathologic subtype and grading for each lesion. Indeed, despite the small amount of tissue analysed, haematoxylin and eosin-stained slides were blindly reviewed by two pathologists in order to provide additional information that could correlate with the radiological features. A complete histological assessment of each lesion was performed, which included the presence of necrosis, haemorrhages, desmoplasia, lymph vascular invasion, and finally the presence of tumour-infiltrating lymphocytes.

### Statistical analysis

Statistical spectral analyses were performed using Mann-Whitney *U* test (Matlab R2021b) and *p* values lower than 0.05 were considered significant. The power analysis was performed with G-power 3.1.9.7 software (https://www.psychologie.hhu.de/arbeitsgruppen/allgemeine-psychologie-und-arbeitspsychologie/gpower) (Supplementary Fig. 1).

## Results

### Patients

Seventeen patients with histologically proven CC were included in the study. Of these, eleven were staged at baseline MRI as LACC and treated with neoadjuvant chemotherapy; the remaining six were directly managed with radical surgery. Five of eleven were excluded from subsequent analyses (three were lost to follow-up and two had a nondiagnostic spectrum because of a signal-to-noise ratio < 4). Thus, our final sample consisted of six patients, of whom three were GR and three PR-or-NR.

Patients’ mean age was 54.8 ± 16.3 years (mean ± standard deviation). Maximum tumour diameter was 75.0 ± 13.4 mm (mean ± standard deviation). Four patients had squamous tumour histotype, and two had adenocarcinoma. Concerning tumour grading, five were grade G2, and only one is grade G3; four had positive pelvic lymph nodes on MRI (FIGO 2018 stage IIIc1r). A summary of patients’ characteristics is presented in Table 2.

**Table 2:** Patients’ characteristics

	Population (*n* = 6)	GR group (*n* = 3)	PR-or-NR group (*n* = 3)
Age (years)	54.8 ± 16.3^a^	34, 74, 43	64, 46, 65
Maximum tumour diameter (mm)	75.0 ± 13.4^a^	70, 58, 96	70, 71, 85
FIGO stage			
IIb	0	0	0
IIIc1	4	3	1
IIIc2	1	0	1
IVa	1	0	1
Histotype			
Squamous carcinoma	4	1	3
Adenocarcinoma	2	2	0
Grading			
G1	0	0	0
G2	5	2	3
G3	1	1	0

Unless otherwise noted, data are numbers of patients

*FIGO* International Federation of Gynecology and Obstetrics, *GR* good responder, *PR-or-NR* partial responder or nonresponder

^a^Mean ± standard deviation

### Spectral analysis

**Fig. 1:**
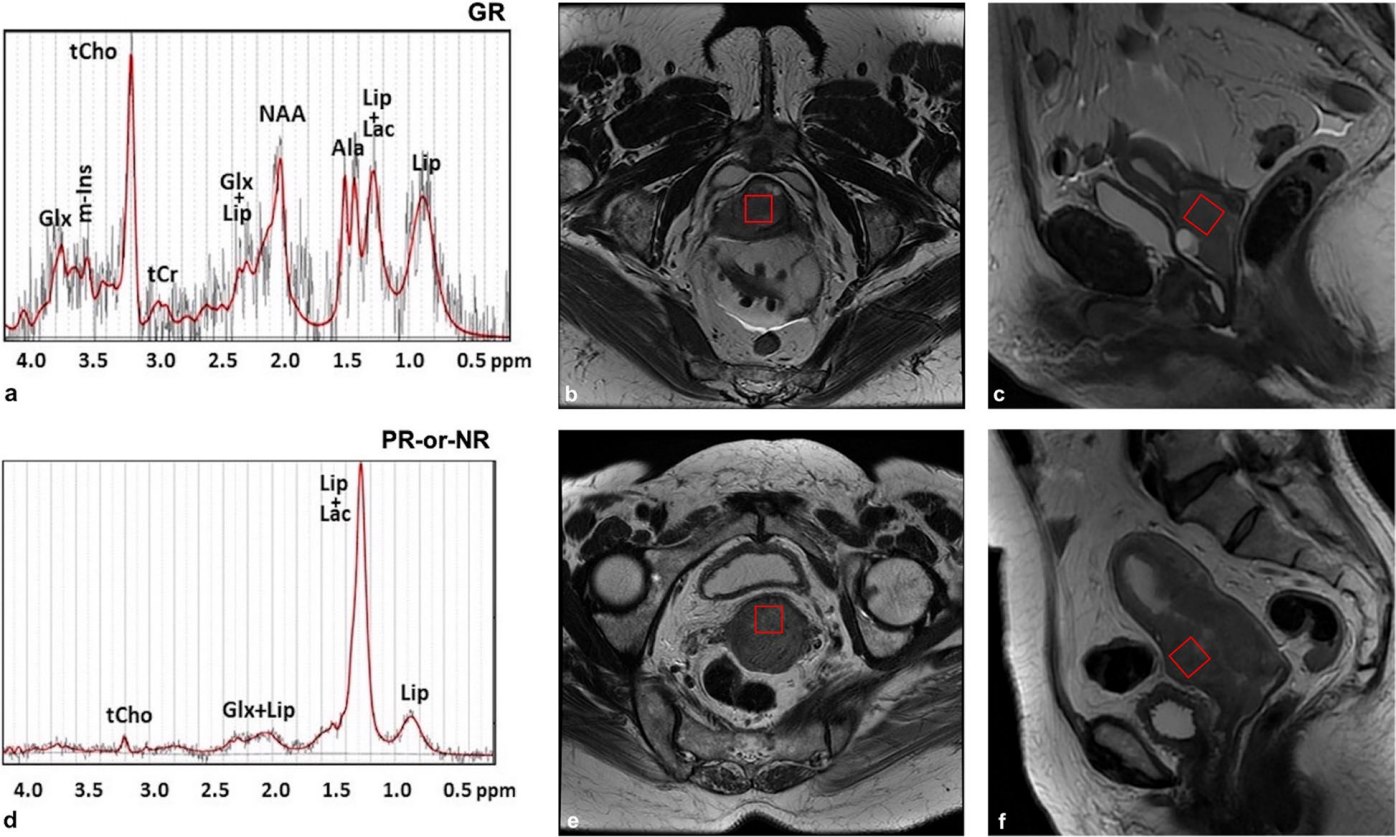
Representative spectra (echo time 30 ms) of the two groups: (**a**) low and (**d**) high lipid level, corresponding to good responder patients (GR) and partial or nonresponder patients (PR-or-NR), respectively. Peak assignments: Glx, Glutamine plus glutamate; m-Ins, Myo-inositol; tCho, GPC plus PCho or total choline; tCr, Creatine plus phosphocreatine; Ala, Alanine; Lip, Lipids; Lac, Lactate. T2-weighted paraaxial (**b**, **e**) and sagittal (**c**, **f**) images of the two cases

In the spectra obtained from our uterine cervical tumours, it was possible to identify the signals of Glx, m-Ins, tCho, tCr, NAA, Ala, Lac, and Lip, as shown in Fig. 1a. Quantification was obtained for those metabolites who satisfy the condition of Cramer-Rao lower bounds less than 20% and therefore for tCho, Glx, NAA, and Lip. Short TE (28 ms) spectra presented a different metabolic pattern in the two populations GR and NR/PR. In particular, as a first result, we observed that the mean Lip value in GR patients (100 ± 50 mM) was significantly lower than in PR-or-NR patients (270 ± 50 mM) (*p* = 0.040). Representative spectra are shown in Fig. 1a (low lipid level, L) and Fig. 1d (high lipid level, H). Long TE (144 ms) spectra analysis showed absence or low level of Lac at 1.3 ppm, thus excluding lipid overestimation in the quantitative analysis.

**Fig. 2:**
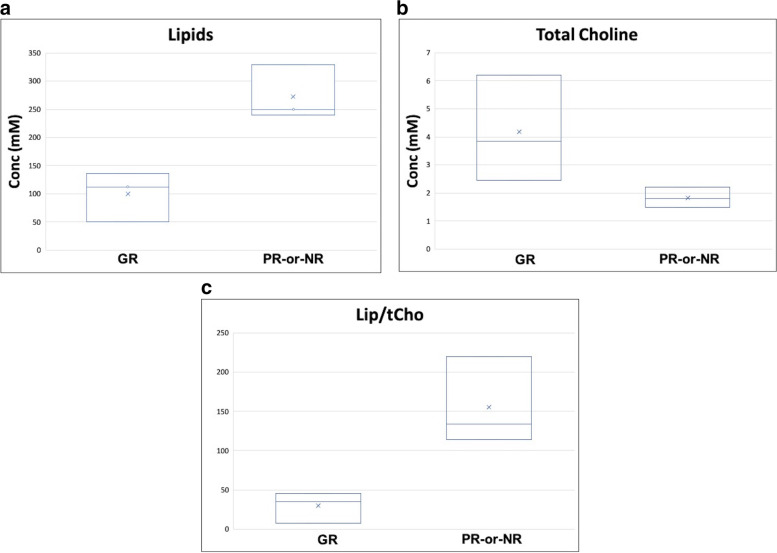
Boxplot of lipid signal, resonating at 1.3 ppm (**a**) total choline (tCho) signal (**b**) and lipids/tCho (Lip/tCho) ratio (**c**) in the two groups: good responder patients (GR) on the left and partial or nonresponder patients (PR-or-NR) on the right

Moreover, a significant difference was also observed in tCho distribution. In the GR group, the mean tCho peak value (4.2 ± 1.9 mM) was significantly higher than in the PR-or-NR group (1.8 ± 0.4 mM) (*p* = 0.040). Finally, we also observed a significant difference in the Lip/tCho ratio with higher values in PR-or-NR patients (30 ± 20) than in GR patients (160 ± 60) (*p* = 0.040). Figure 2 shows the box plots of the signals of Lip (**a**), tCho (**b**) and of the ratio Lip/tCho (**c**), and Table 3 summarises the data obtained.

**Table 3:** *In vivo* quantitative ^1^H magnetic resonance spectroscopy determinations of metabolite concentrations in spectra of high (H, *n* = 3) and low or intermediate lipid content (L, *n* = 3) in uterine cervical cancers

	H	L	*p* values
Lip at 1.28 ppm	270 ± 50	100 ± 50	0.040*
Glx	14 ± 5	12 ± 8	0.331
NAA	3.8 ± 3.5	6.2 ± 2.2	0.877
tCho	1.8 ± 0.4	4.2 ± 1.9	0.040*
Lip/tCho	160 ± 60	30 ± 20	0.040*

Metabolite concentrations are expressed in mM

*Glx* Sum of glutamate (Glu) and glutamine (Gln), *Lip* Lipids, *Lip/tCho* Ratio between lipids and total choline, *NAA N*-acetylaspartate, *tCho* Total choline

*Indicates statistically significant values (Mann-Whitney *U* test)

No significant correlation was observed between spectral pattern and clinical and histological data, in particular, Lip, tCho, and Lip/tCho values did not show significant differences when varying age, tumour diameter, histotype, grading, and lymph nodes’ presence on MRI.

## Discussion

As observed in our study, ^1^H-MR spectroscopy at 3.0 T can be successfully used to probe the molecular profile of uterine cervical tumours *in vivo*. The use of 32-channel receiver coil made it possible to obtain *in vivo* spectra from the tumour in a reasonable time (less than 5 min).

By acquiring short TE spectra (TE = 30 ms), we detected and quantified lipid resonances (the triglyceride methylene −CH_2_ signal resonating at 1.28 ppm and the methyl −CH_3_ signal resonating at 0.9 ppm) in all our tumours. Previous studies performed on patients or on *ex vivo* biopsies used alterations in fatty acid metabolism to discriminate between preinvasive and invasive tumours or to differentiate between cancer and healthy control groups [20, 25, 26].

Lipids are a group of biomolecules that constitute the structural basis of biological membranes and function as energy source and signalling molecules [27]. Many roads lead to lipid accumulation in cancer, and metabolic reprogramming is one of them, which has been widely observed during cancer development to confer cancer cells the ability to survive and proliferate. Together with the well-known ‘Warburg effect’ (aerobic glycolysis, even when the ambient oxygen supply is sufficient), deregulated anabolism/catabolism of fatty acids and amino acids (mainly glutamine, serine, and glycine) has been identified to support cancer cell growth [28]. Tumour cells prefer the fatty acid oxidation pathway as a source of energy, which can come from either external or newly formed fatty acids, which are oxidised and stored as lipid droplets in the tissue. Fatty acid oxidation has been proven to protects cancer cells from treatment induced apoptosis by increasing mitochondrial membrane lipids [29].

Previous studies applied *ex vivo*^1^H-MRS to CC biopsies at 8.5 T and detected differences in lipid signal with both single voxel techniques and chemical shift microimaging [25, 30]. The authors could clearly distinguish preinvasive from invasive cancer evaluating the intensity of the lipid signal (detected by single voxel ^1^H-MRS) or on the intensity of the bright spot obtained in the lipid maps of chemical shift microimaging of the biopsies. The higher lipid level was found in the invasive cancers. Moreover, a recent *in vivo* study performed at 7 T on uterine CC identified poorly differentiated tumours because they showed elevated ratios between two fatty acid groups (the first composed by α-carboxyl and α-olefinic groups resonating at about 2.1 ppm over the methyl group resonating at 1.3 ppm) when compared with the well-differentiated tumour or normal tissue [18]. In other words, more unsaturated fatty acids were found in poorly differentiated tumours. These results are in agreement with previous published MRS studies at lower magnetic field strengths, where fatty acid signals were observed in uterine CC [17–22].

Unfortunately, 7-T system which allow the distinction of the lipid resonances at 2.1 ppm from other metabolites (such as Glx or NAA) are not common in clinical settings, making this application limited to few research institutes. Our quantitative protocol, which resemble an absolute quantification (except for the eventual T1 alterations), can be widely applied and the results can be compared to different centres with different systems. The limitation of using the methylene 1.28 ppm peak for lipid quantification may cause lipid overestimation as it overlaps with the methyl resonance of lactate, which also appears at 1.3 ppm and could be present in tumours. In our protocol, we added an extra acquisition at longer TE (144 ms) in order to detect (as a negative doublet) and eventually quantify the presence of lactate. In our spectra, Lac have been observed in only few spectra and in these in a small amount.

The tCho peak has also been considered in previous studies for discrimination between benign and malignant cervical lesions with contradicting results [17]. It includes the complex of choline compounds (choline, PCho, and glycerophosphocholine) and has been found elevated in several tumours. In fact, aberrant choline metabolism, characterised by an increase in total choline-containing compounds, is a metabolic hallmark of carcinogenesis and tumour progression. It arises from alterations in the metabolic enzymes that control the biosynthesis and catabolism of phosphatidylcholine. As a result of these metabolic changes, high PCho and tCho content is often observed in actively growing tumours. With limitations due to poor sensitivity, tCho have been identified as diagnostic biomarkers of malignancy in prostate, breast and brain cancer and as predictors of tumour aggressiveness in breast cancer [31–34]. These authors found an association of tCho levels with higher grading, higher Ki-67 value and large-sized lesions. They also found a significant association between the presence of tCho and the absence of central necrosis. Although limited to a small number of patients, our data show an increase in tCho in GR patients and may suggest an inverse correlation between lipids and tCho. All these data are in agreement with a better response of low-necrosis and therefore better-perfused tumours, as previously assessed in other gynaecological cancers [9]. Other signals were also present in our sample of CC (*i.e.*, Glx and, in some cases, NAA) but their quantification did not reveal differences that could predict a favourable prognosis.

Notably, ^1^H-MRS is a noninvasive method and it is easily accessible to several centres using a well-tolerated procedure, and the addition of spectroscopy sequences to the standard MRI protocol led to an average time extension of a few minutes, which would not negatively affect clinical practice.

Our study had limitations. The first is the very limited number of patients included in the quantitative analysis. Nevertheless, power analysis confirmed that, because of the substantial difference found between the Lip signal values of GR and PR-or-NR, the number of patients analysed is sufficient to obtain significant and encouraging results in this preliminary study (Supplementary Fig. 1). Further analysis with a large cohort of patients is warranted. Second, our data are based on tumour lesions larger than 58 mm, a size that ensures that analysed volumes completely cover the entire tumour volume. Uterine CCs of this size can be heterogeneous, but MRS provides an average signal arising from the entire voxel. Moreover, application of the quantitative protocol should ensure detection of high lipid content even if this amount does not come from the entire volume, suggesting the presence of nonresponder-like regions within the tumours.

In conclusion, our data showed the ability to noninvasively detect and quantify lipid profiles in CCs by using ^1^H-MRS at a magnetic field widely used in clinical settings (3 T). For the first time, it was observed that lipid level could be associated with tumour prognosis (the higher the lipid signal, the worse the prognosis). *In vivo*^1^H-MRS may have the potential as a prognostic factor of CC in a noninvasive manner to help clinical management.

## Supplementary Information

**Additional file 1: Figure S1.** Power analysis (or sample size calculation) for the Mann-Whitney test of the lipid signal.

## Data Availability

The datasets used and/or analysed during the current study are available from the corresponding author on reasonable request.

## Abbreviations

^1^H-MRS: Proton magnetic resonance spectroscopy
Ala: Alanine
CC: Cervical cancer
FIGO: International Federation of Gynecology and Obstetrics
Gln: Glutamine
Glu: Glutamate
Glx: Glu + Gln
GPC: Glycerophosphorylcholine
GR: Good responders
Ins: Myo-inositol
Lac: Lactate
LACC: Locally advanced cervical cancer
Lip: Lipids
MRI: Magnetic resonance imaging
NAA: *N*-acetylaspartate
PCho: Phosphorylcholine
PR-or-NR: Poor responders or nonresponders
tCho: Total choline (i.e. GPC+PCho)
TE: Echo time
