# Supplementary material for: The contribution of the 1H-MRS lipid signal to cervical cancer prognosis: a preliminary study
Source: Eur Radiol Exp. 2022 Oct 3;6:47. doi: 10.1186/s41747-022-00300-1 (PMC9527268; doi:10.1186/s41747-022-00300-1)
Supplement: Supplementary file 1 — Additional file 1: Figure S1. Power analysis (or sample size calculation) for the Mann-Whitney test of the lipid signal. [file 41747_2022_300_MOESM1_ESM.pdf]

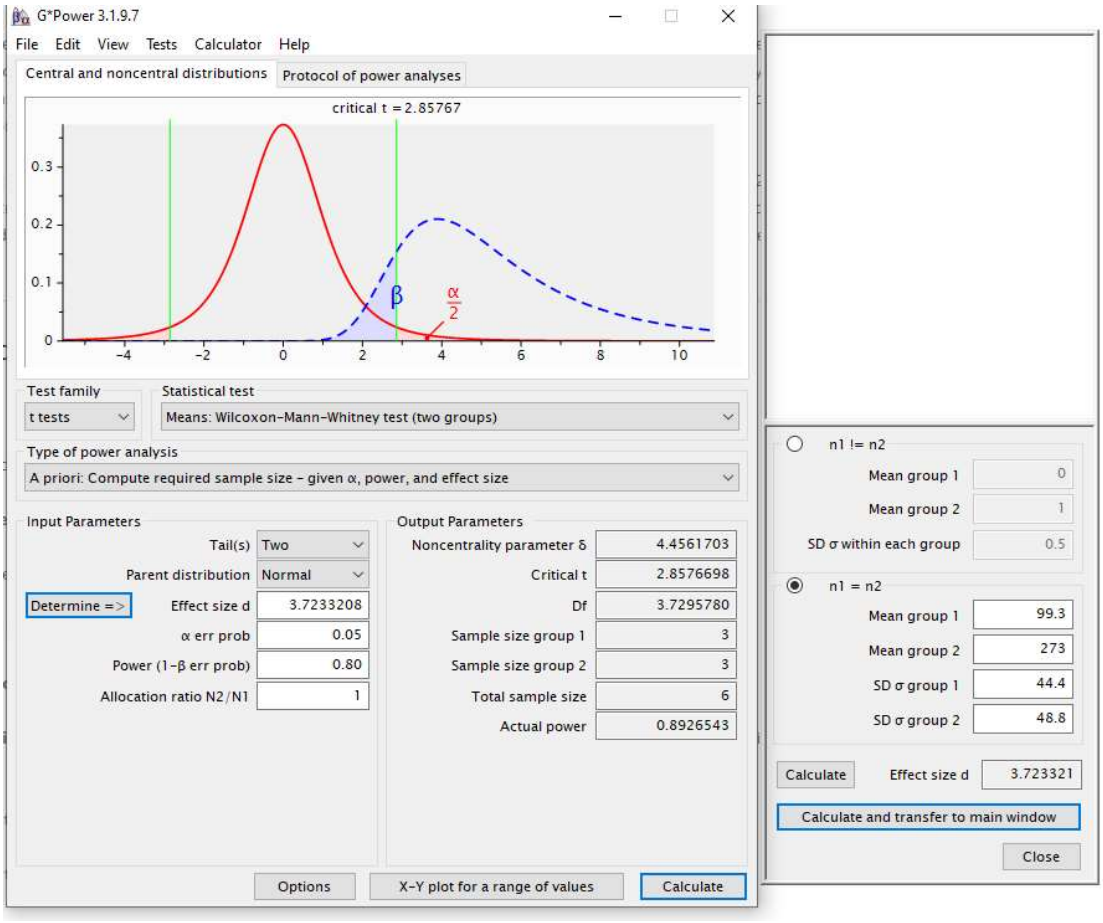

Supplementary Figure 1: Power analysis (or sample size calculation) for the Mann-Whitney test of the lipid signal.
